# Molecular Descriptors as a Facile Tool toward Designing Surface-Functionalized Nanoparticles for Drug Delivery

**DOI:** 10.1021/acs.molpharmaceut.1c00940

**Published:** 2022-03-22

**Authors:** Sourav Bhattacharjee

**Affiliations:** School of Veterinary Medicine, University College Dublin (UCD), Belfield, Dublin 4, Ireland

**Keywords:** molecular descriptors, polymer brush, Tanimoto coefficient, nano-DDS, SwissADME, ChemMine tools, drug target prediction, targeted delivery, hierarchical clustering

## Abstract

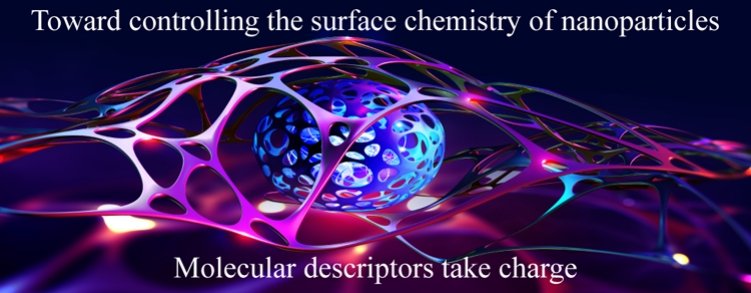

Modulating the surface chemistry of nanoparticles, often by grafting hydrophilic polymer brushes (e.g., polyethylene glycol) to prepare nanoformulations that can resist opsonization in a hematic environment and negotiate with the mucus barrier, is a popular strategy toward developing biocompatible and effective nano-drug delivery systems. However, there is a need for tools that can screen multiple surface ligands and cluster them based on both structural similarity and physicochemical attributes. Molecular descriptors offer numerical readouts based on molecular properties and provide a fertile ground for developing quick screening platforms. Thus, a study was conducted with 14 monomers/repeating blocks of polymeric chains, namely, oxazoline, acrylamide, vinylpyrrolidone, glycerol, acryloyl morpholine, dimethyl acrylamide, hydroxypropyl methacrylamide, hydroxyethyl methacrylamide, sialic acid, carboxybetaine acrylamide, carboxybetaine methacrylate, sulfobetaine methacrylate, methacryloyloxyethyl phosphorylcholine, and vinyl-pyridinio propanesulfonate, capable of imparting hydrophilicity to a surface when assembled as polymeric brushes. Employing free, Web-based, and user-friendly platforms, such as SwissADME and ChemMine tools, a series of molecular descriptors and Tanimoto coefficient of molecular pairs were determined, followed by hierarchical clustering analyses. Molecular pairs of oxazoline/dimethyl acrylamide, hydroxypropyl methacrylamide/hydroxyethyl methacrylamide, acrylamide/glycerol, carboxybetaine acrylamide/vinyl-pyridinio propanesulfonate, and sulfobetaine methacrylate/methacryloyloxyethyl phosphorylcholine were clustered together. Similarly, the molecular pair of hydroxypropyl methacrylamide/hydroxyethyl methacrylamide demonstrated a high Tanimoto coefficient of >0.9, whereas the pairs oxazoline/vinylpyrrolidone, acrylamide/dimethyl acrylamide, acryloyl morpholine/dimethyl acrylamide, acryloyl morpholine/hydroxypropyl methacrylamide, acryloyl morpholine/hydroxyethyl methacrylamide, carboxybetaine methacrylate/sulfobetaine methacrylate, and glycerol/hydroxypropyl methacrylamide had a Tanimoto coefficient of >0.8. The analyzed data not only demonstrated the ability of such *in silico* tools as a facile technique in clustering molecules of interest based on their structure and physicochemical characteristics but also provided vital information on their behavior within biological systems, including the ability to engage an array of possible molecular targets when the monomers are self-assembled on nanoparticulate surfaces.

## 1 Introduction

The use of nanoparticles (NPs) as drug delivery systems (DDSs) has increased considerably in the past few decades.^1^ Such growth in theranostic applications of NPs is further catalyzed by the unique set of materialistic properties (e.g., magnetism, enhanced surface reactivity, and fluorescence) that provide NPs an edge over larger particles, including microparticles.^2−6^ A minuscule size also ensures accessibility for the NPs to those sites in the human body that are otherwise difficult for microparticles to reach due to size constraints. Moreover, the high reactivity of NPs opens a wide range of opportunities for bioconjugation, for example, with homing peptides like Arg-Gly-Asp^7^ or ligands like folic acid^8^ to target pathologic sites in the body, such as tumors known to over-express folate receptors or integrins on the walls of leaky intratumoral vasculature.^9^

**Figure 1:**
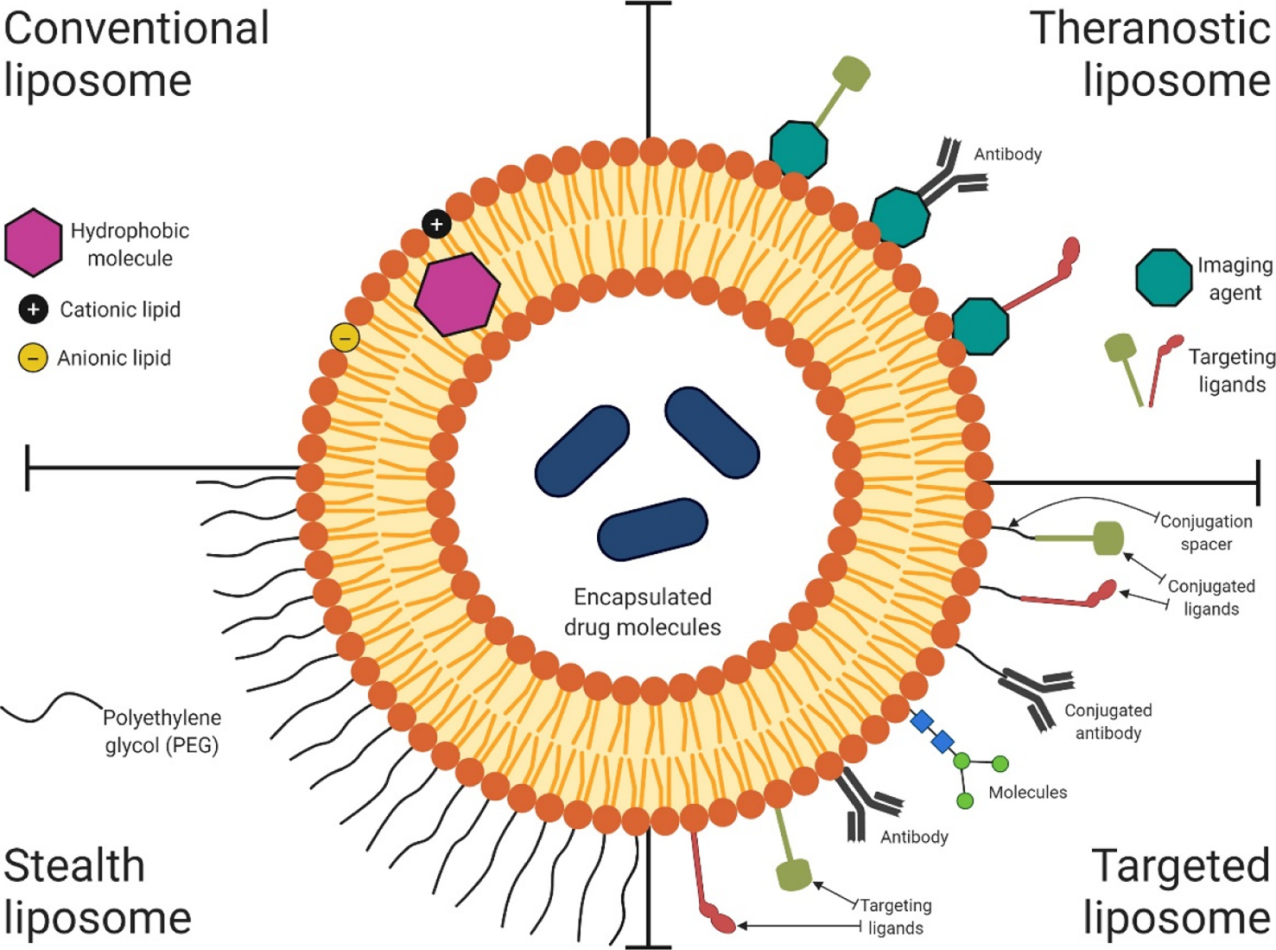
Scheme showing a liposomal nano-DDS with encapsulated drugs in its core and lipid bilayer. The surface chemistry of such nano-DDSs is manipulated deliberately to link a wide range of theranostic molecules, either by surface grafting or by adsorption, to develop stealth and targeted nanoformulations.

A key message that distills out upon scrutiny of the literature on nano-DDSs is the importance of surface chemistry in nano-bio interactions.^10^ It is established that the nanoparticulate surface chemistry determines its behavior within biological systems (Figure 1), including toxicity, cellular uptake, tissue targeting, and importantly, release kinetics.^11^ For example, there is compelling evidence of higher toxicity and cellular uptake of cationic NPs than their anionic counterparts.^12^ Similarly, an augmented surface polarity, often attained by surface grafting of hydrophilic blockchains, such as polyethylene glycol (PEG), has emerged as an attractive strategy toward developing NPs that can negotiate with physiological barriers,^13^ such as the gut mucus layer.^14^

To a certain extent, the concept of coating NP surfaces with hydrophilic molecules while designing mucopermeative nano-DDSs was inspired by the examples of viruses. It was noted that viruses, such as poliovirus, Norwalk virus, or respiratory syncytial virus, can freely diffuse through the mucus barrier (e.g., gut, eyes, and respiratory and genital tracts).^15^ Given that mucus has evolved into a formidable physiological barrier over epochs, such unprecedented mucopermeation demonstrated by the viruses has intrigued researchers, although the mechanism is still unclear. It is known that viruses exhibit a high surface density of polar groups, and there is a growing consensus that the cloak of polar groups surrounding the viruses causes a *hydrophilic brush effect*,^16^ where it drags a meniscus of water with it while permeating the mucus.^17^ As a result, the mucoadhesion of the viral particles is deterred, and viral mobility inside mucus can proceed (almost) unhindered.

Mucin, a zwitterionic fibrillar glycoprotein biopolymer, forms most of the mucus’s solid components and supports its characteristic hydrogel form.^18^ Mucin fibers form a complex network with interspersed pores that act as a sieve and prevent particulate materials from permeating unless smaller than the pores.^19^ However, these pores are not static, and their dimensions keep fluctuating (10–500 nm) based on multiple factors, including pathophysiological state, site, degree of desiccation, or oxidation.^20^ Moreover, the mucin fibers harbor cationic and anionic sites arranged in tandem that, based on electrostatic interactions, act as traps for charged NPs. Thus, upon meeting mucus, NPs, unlike viruses, typically adhere and get sloughed off with the superficial mucus layers recycled within the body, for example, every 3–4 h in the case of gut mucus.^21^

Another challenge with nano-DDSs is their rapid filtration from blood after intravenous administration—widely known as the *accelerated blood clearance* phenomenon.^22,23^ Such brisk sieving is induced by the activation of the reticuloendothelial system in the body, releasing macrophages to phagocytose the NPs circulating in the bloodstream.^24^ Instant coating of the NPs with opsonins (e.g., albumin and fibrinogen) present in the blood by surface adsorption initiates the cascade of macrophagic invasion.^25^ Rapid phagocytosis ensures that a significant fraction, sometimes more than 90%, of the injected NPs are withdrawn from the blood and sequestered into the liver, lungs, spleen, and bone marrow.^26^ Hence, the administered nano-DDSs, despite mechanisms promoting accumulation into tumor sites, such as the *enhanced permeation and retention* effect,^27^ fail to achieve an adequate therapeutic concentration inside a tumor.

Imparting surface polarity to the NPs is known to deter mucoadhesion, facilitate the mobility of such stealth NPs in a mucus mesh, and resist the opsonization of NPs once introduced into the blood.^28^ Thus, the plasma half-life of the nano-DDSs is prolonged. The current strategy of achieving that relies fundamentally on modulating the surface chemistry via conjugation, either by covalent bonding or by surface adsorption. PEGylation,^29^ achieved mostly through surface conjugation, elucidates the hydrophilic brush effect and induces mucopermeation. However, as a molecule, PEG is biopersistent and can induce allergic reactions in the host body.^30−33^ Moreover, PEGylation is not a facile technique and, at times, requires harsh reaction conditions that might be deleterious toward a labile drug payload.

Currently, a search is ongoing to find suitable replacements of PEG that can be used for surface functionalization of NPs. For example, a series of hydrophilic polymers like polyoxazoline (POZ), poly(*N*-vinylpyrrolidone) (PVP), polyglycerol (PG), and polyacrylamide (PAA) has been probed.^34^ Similarly, natural [e.g., dextran, heparin, and polysialic acid (PSA)] and zwitterionic [e.g., poly(carboxy betaine) (pCB), poly(sulfobetaine) (pSB), and phosphobetaine] polymers have also been explored. However, such investigations have led to the challenge of screening and shortlisting the right candidate(s) from an extensive list of low molecular weight molecules or ranking a library of surface ligands based on their hydrophilicity while taking biocompatibility into account.

An interesting way to make such a choice is to rely on the molecular descriptors that signify a molecule’s physicochemical attributes^35^—in entirety or partially. With advanced *in silico* tools, a wide range of 1D, 2D, 3D, and 4D molecular descriptors can be extracted from the analyses of molecular structures in the form of numerical readouts. Fortunately, some Web-based applications, such as SwissADME (http://www.swissadme.ch/), are free, simple, intuitive, and user-friendly and run with an integrated platform for drawing chemical structures or inserting a simplified molecular-input line-entry system (SMILES) of molecules.^36^ If necessary, more advanced molecular simulation tools, such as open-source PaDEL, which calculates 1875 descriptors (1444 1D and 2D descriptors plus 431 3D descriptors) along with 12 molecular fingerprints, can be employed.^37^

Free Web-based computational platforms to determine thousands of molecular descriptors, such as ChemDes (http://www.scbdd.com/chemdes/; Computational Biology & Drug Design Group, School of Pharmaceutical Sciences, Central South University, China), are also emerging.^38^ These integrated Web platforms can provide a wealth of information about molecules in spreadsheets that can further be used for clustering analyses. Determining molecular similarity based on the Tanimoto coefficient (also known as the Jaccard coefficient) is an interesting way of comparing a library of molecules.^39^ Taken together, the data points form an integral part of cheminformatics and quantitative structure–activity relationship-based studies with implications for designing nano-DDSs.

This paper will demonstrate the utility of converging data analytics to a data set of molecular descriptors and reveal the clustering of molecules based on their physicochemical attributes. Additionally, the process will demonstrate how this technique can be useful in screening a library of suitable low molecular weight drug-like molecules and then isolating the best candidates for follow-up. To do so, a series of monomers that have been prioritized so far for surface functionalizing of NPs will be clustered based on their molecular descriptors. Finally, the discourse will identify the strengths and weaknesses of such a computational approach before prioritizing some future directions for nanomedicine research from the perspective of surface functionalization guided by such *in silico* tools.

## 2 Experimental Section

### 2.1 Selection of Monomers

Based on the literature on grafted polymers on NPs to facilitate mucodiffusion and extension of blood circulation time, a library of 14 monomers depicting the repeating structural blocks of the polymeric chain was developed and marked as oxazoline (OZ), acrylamide (AA), vinylpyrrolidone (VP), glycerol (Gly), acryloyl morpholine (AcM), dimethyl acrylamide (DMA), hydroxypropyl methacrylamide (HPMA), hydroxyethyl methacrylamide (HEMA), sialic acid (SA), carboxybetaine acrylamide (CBAA), carboxybetaine methacrylate (CBMA), sulfobetaine methacrylate (SBMA), methacryloyloxyethyl phosphorylcholine (MPC), and vinyl-pyridinio propanesulfonate (VPPS).

### 2.2 Determination of the Molecular Descriptors

Chemical structures of the monomers were drawn in ChemDraw Ultra (Version: 12.0.2.1076; PerkinElmer, Inc., Waltham, MA, USA) and saved as ChemDraw (.cdx) files. Later, the structures were uploaded in the SwissADME user interface (www.swissadme.ch)—a free Web tool developed and maintained by the Swiss Institute of Bioinformatics—to generate the SMILES and evaluate the pharmacokinetics, drug-likeness, and medicinal chemistry of the molecules. Upon loading the molecular structures of the monomers, the SwissADME suite was run to obtain a set of molecular descriptors asserting the physicochemical properties (e.g., molecular weight, number of heavy atoms, the fraction of *sp^3^* hybridized carbon atoms, number of hydrogen bond acceptors, donors, molar refractivity, and total polar surface area in Å^2^), lipophilicity (e.g., log *P*_o_/*w*), solubility (e.g., log *S*), pharmacokinetics [e.g., absorption across the gastrointestinal tract and blood–brain barrier (BBB), suitability as a substrate of the efflux transporter P-glycoprotein (P-gp), and ability to inhibit cytochrome P (CYP) enzymes like CYP1A2], drug-likeness (based on the scales of Lipinski, Ghosh, Veber, Egan, and Muegge with bioavailability), and medicinal chemistry (lead-likeness and synthetic accessibility). The obtained data set for each monomer was collated as spreadsheets for further analyses.

### 2.3 Identification of Molecular Targets

The molecular SMILES of the 14 monomers were uploaded into the online SwissTargetPrediction suite (http://www.swisstargetprediction.ch/) linked to the SwissADME Web application followed by the selection of *Homo sapiens* (humans) as target species and running the program.^40,41^ Target molecules with the highest probabilities were noted.

### 2.4 Calculation of the Tanimoto coefficient

The Tanimoto coefficient was determined by the ChemMine tools (https://chemminetools.ucr.edu/)—a free Web-based application developed by researchers from the University of California Riverside (CA, USA)—to analyze and cluster small molecules.^42^ The molecular structures of the 14 monomers were fed into the system by inserting SMILES, followed by activating the Similarity Workbench suite within the application. The coefficient was calculated by measuring *c*/(*a* + *b* + *c*), where *c* denotes the number of features shared by both the molecules, whereas *a* and *b* represent unique features present in each molecule. The monomers were compared in pairs, and the Tanimoto coefficient was measured based on the maximum common substructure algorithm.^43^

### 2.5 Hierarchical Clustering and Generation of Heatmap Based on Tanimoto coefficient

A dendrogram based on the agglomerative hierarchical clustering of the 14 monomers/repeating units was generated using OriginPro 2015 software (OriginLab Corporation, Northampton, MA, USA) under pre-decided settings (clustering method: group average; distance type: Euclidean; find clustroid by: sum of distances). Furthermore, a heatmap was developed after plotting the Tanimoto coefficient data matrix in OriginPro 2015.

## 3 Results

### 3.1 Choice of Monomers

The details of the 14 monomers chosen are shown in Table 1.

**Table 1:**
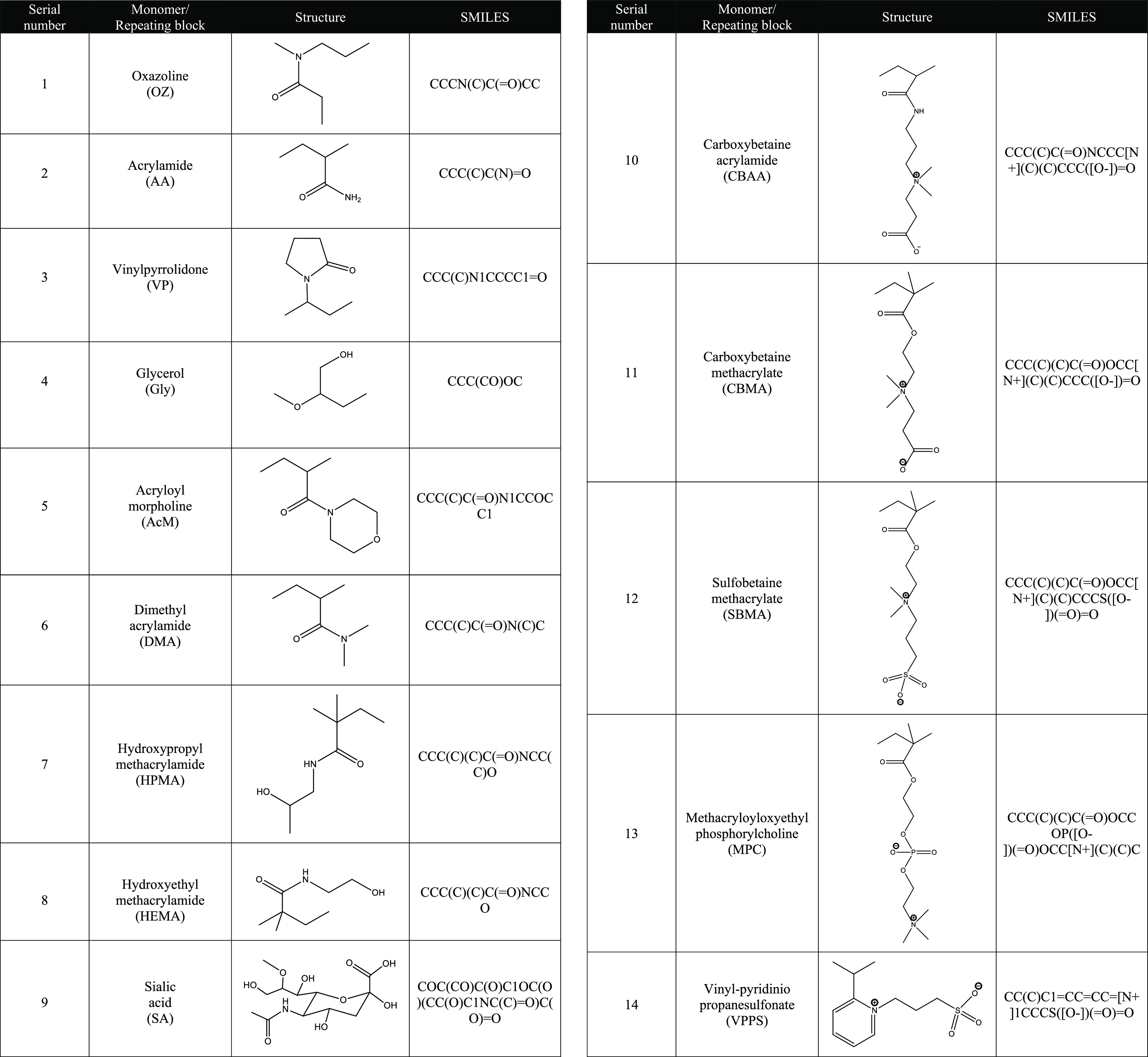
Molecular Structures and SMILES of the 14 Monomers

### 3.2 Hierarchical Clustering of the Monomers

**Figure 2:**
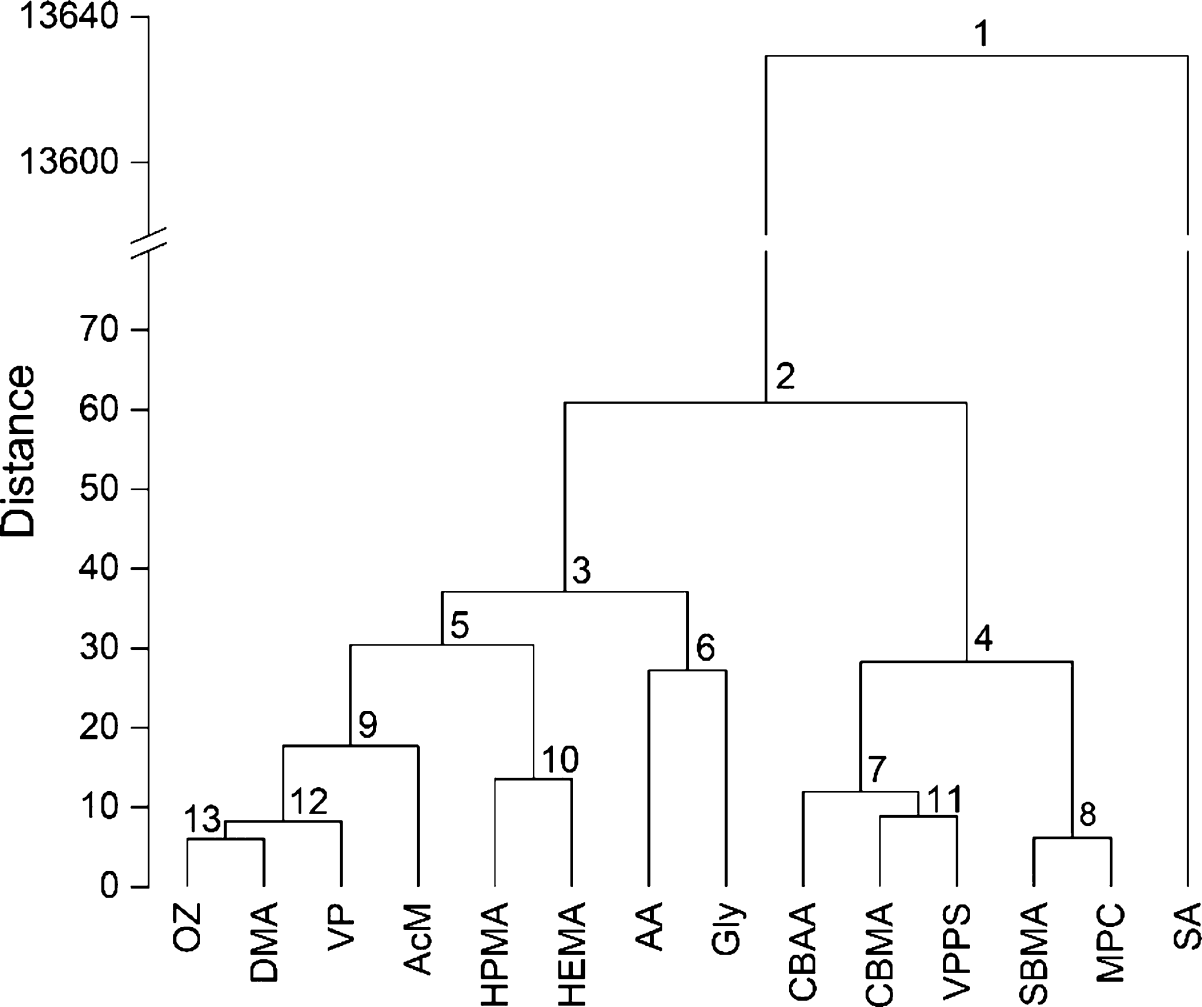
Dendrogram showing the hierarchical clustering of 14 monomers based on their molecular descriptors. The clades are marked numerically (1–13).

The data set of molecular descriptors (Section S1) was subjected to hierarchical clustering, and a dendrogram was generated (Figure 2). The SA monomer was grouped in a different clade than the remaining 13 monomers (clade 2) due to its characteristic pyranose ring, whereas the other monomers were (mostly) linear. The 13 monomers were grouped under two clades comprising eight (clade 3) and five (clade 4) monomers. Further analyses clustered the pairs OZ and DMA (clade 13), HPMA and HEMA (clade 10), AA and Gly (clade 6), CBMA and VPPS (clade 11), and SBMA and MPC (clade 8) together.

### 3.3 Molecular Similarity of the Monomers Based on the Tanimoto Coefficient

**Figure 3:**
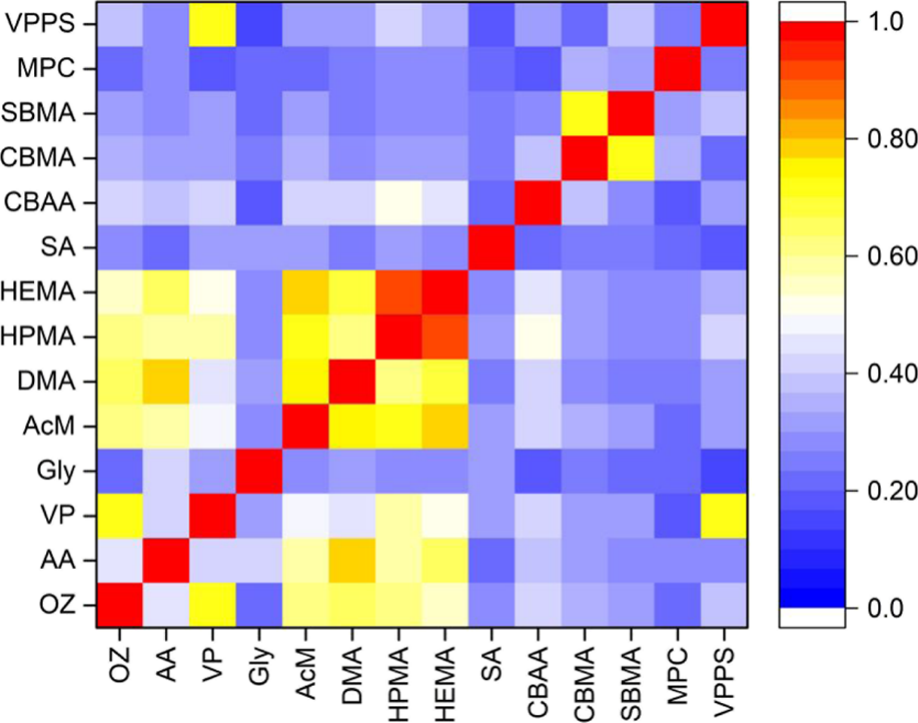
Heatmap showing the array of 14 monomers based on their molecular similarity determined by the Tanimoto coefficient. The color scale is provided as a sidebar.

The molecular similarity plotted as a heatmap based on the Tanimoto coefficient data matrix (Section S2)—derived from a paired comparison of the 14 monomers—is shown in Figure 3. Considerable molecular similarity (Tanimoto coefficient ≥ 0.6) was noted between the following pairs: AA and DMA, VP and VPPS, AcM and DMA, AcM and HPMA, AcM and HEMA, and CBMA and SBMA.

### 3.4 Target Prediction Study

Target prediction study revealed a library of enzymes and receptor proteins as interactive and binding sites for these monomers. A list of identified molecular targets and predictions on absorption across the gut and BBB while the ability to act as a substrate for P-gp efflux transporter proteins are given in Table 2.

**Table 2:** Predicted Abilities of the Monomers toward Gut Absorption, Act as a BBB Permeant or P-gp Substrate, and the Various Molecular Targets It Can Bind^a^

monomer abbreviation	gut absorption	BBB permeant	P-gp substrate	molecular targets
OZ	high	yes	no	oxidoreductase, GPCR, hydrolase
AA	high	yes	no	oxidoreductase, protease
VP	high	yes	no	oxidoreductase, protease, hydrolase
Gly	high	no	no	dioxygenase, hydrolase
AcM	high	yes	no	protease, CYP450, kinase
DMA	high	yes	no	oxidoreductase, GPCR
HPMA	high	yes	no	transporter proteins, GPCR, CYP450, kinase
HEMA	high	yes	no	CYP450, GPCR, kinase
SA	low	no	yes	cytosolic proteins, kinase, voltage-gated ion channel
CBAA	high	no	no	membrane and nuclear receptors, GPCR, transcription factor, phosphodiesterase, phosphatase
CBMA	high	no	yes	oxidoreductase, GPCR, nuclear factors, secreted proteins, phosphatase, protease
SBMA	high	no	yes	GPCR, protease, kinase, voltage-gated ion channel, nuclear receptor, phosphatase
MPC	high	no	yes	oxidoreductase, protease, GPCR, kinase, CYP450, voltage-gated ion channel
VPPS	high	yes	no	CYP450, protease, kinase

a Abbreviations: CYP, cytochrome P; GPCR, G-protein-coupled receptor.

## 4 Discussion

Once introduced to a biological medium, NPs undergo a complex and often unpredictable cascade of interactions where the surface chemistry of NPs appears to be a major player.^44^ Therefore, it is important to gain control over the surface chemistry of NPs, especially those developed as DDSs. However, acquiring knowledge on the suitability and efficacy of surface functionalizing ligands is time-consuming, with no rule of thumb to guide. Besides, failure trying with a particular ligand risks wastage of funding, human resources, and above all, precious animal lives.

Molecular descriptors provide a numerical data set that can address the issue. The availability of free and Web-based platforms like SwissADME and ChemMine tools further adds to the arsenal of researchers. Advancements achieved at designing *in silico* tools enable precise prediction of molecular properties,^45^ including their physicochemical attributes, that are known to play a vital role in determining the behavior of an NP at a nano–bio interface. Surface grafting of polymeric chains is a popular technique to impart surface hydrophilicity, while PEG continues to lead the array of molecules currently employed. The study highlights the potential of a few other low molecular weight molecules in replacing PEG in the future when arranged as polymeric chains.

Molecular descriptors assess the physicochemical attributes based on molecular structure. It is a rational expectation that the same, or at least similar, physicochemical properties will be imparted on an NP surface upon grafting of these molecules in isolation or a polymeric chain. Thus, as elucidated in this discourse, a molecular descriptor-based study on the monomers provides a robust rationale before selecting surface grafting ligands. Fortunately, these tools are easy to use or understand and do not require extensive computational ability, making them optimal for simple exploratory assessments before engaging with wet lab work.

**Figure 4:**
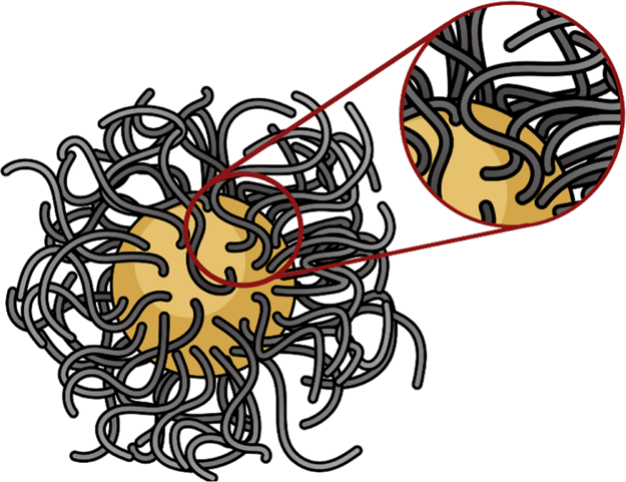
Realistic rendition of a spherical NP grafted with polymeric chains. The inset provides a closer view of an isolated area on the surface, marked with a red circle, demonstrating a surface inclination of the monolayer and proximity for electronic interactions between neighboring chains. The scope for such interactions grows with increased chain lengths.

However, when grafted on a curved surface, a polymeric backbone can differ from its monomers or its isolated and repetitive structural units in electronic properties, while such distinctive behavior is stimulated by the chain’s 3D orientation and biochemical environment (Figure 4).^46^ For example, when grafted on a spherical NP, the polymeric chains are flexible and, rather than vertical, remain inclined at an acute angle to the surface (Figure 4, inset). The surface coverage achieved through the grafting of polymeric chains on an NP is also often heterogeneous, with pockets of high and low densities that affect the spread of polarity across the surface.^47^ Moreover, neighboring chains can interact with each other due to temporospatial proximity. Molecular descriptors, unfortunately, are not able to provide an in-depth reflection of such intra- and inter-chain interactions.

It is crucial to understand the difference between the Tanimoto coefficient and hierarchical clustering based on molecular descriptors. While both these operations try to assess molecular similarity, the results need not be identical. Two molecules with a considerable Tanimoto coefficient (≥0.6) do not necessarily depict a high similarity in the hierarchical clustering method and *vice versa*. An example can be the molecular pair of AA and DMA that had a Tanimoto coefficient of 0.78 and yet was clustered into two distant clades in a dendrogram. On the contrary, CBMA and VPPS were clustered together in the dendrogram despite having a low Tanimoto coefficient (0.21). Another interesting example can be the pair of OZ and VP that, despite sharing molecular similarity, did not demonstrate a high Tanimoto coefficient, such as >0.9. It can be attributed to the difference in molecular configurations: OZ is a linear molecule, while VP harbors a heterocyclic backbone with a carbonyl group attached to it. Thus, the correlation between the Tanimoto coefficient and hierarchical clustering is not linear, although it often does follow a pattern or trend. For instance, HPMA and HEMA were clustered together while also having a high Tanimoto coefficient (0.92).

The subtle discrepancy between the Tanimoto coefficient and hierarchical clustering algorithm arises from the difference in how they are measured. While the Tanimoto coefficient is determined purely based on the molecular structure, the data set of molecular descriptors tries to represent a snapshot of the physicochemical behavior of the molecule. Hence, an analysis of molecules based on molecular descriptors and any similarity identified based on them bears more relevance in drug designing. On the other hand, the Tanimoto coefficient is easy to calculate, requires less computation, and provides a fast screening of a range of smaller molecules based on structural similarity that, at times, may come in handy while designing experiments or selecting chains for surface grafting.

It is worth mentioning that the molecular descriptors included here were mostly 1D and 2D descriptors, while a more detailed study requires 3D descriptors. However, that will be a calculation-intensive and time-consuming process, and at least from an initial screening perspective, hardly provides an edge over 1D and 2D descriptors.^48^ It is also crucial to screen the descriptors as current tools can offer thousands of them. It is prudent to gather the important ones as otherwise the system risks being overwhelmed. Such screening of molecular descriptors will be influenced by the type of molecules being investigated and the information that the *in silico* platform is expected to deliver.

Molecular descriptors also provide a decent understanding of some key features of a grafted molecule, such as its solubility and bioavailability. These parameters are crucial while designing surface-functionalized nano-DDSs, especially for intravenous administration. Predictions are also made on the drug’s potential to be absorbed from the gut, act as a P-gp substrate, or permeate the BBB. Having an initial idea on these essential surface properties of the NPs certainly assists in designing advanced DDSs, especially aiming for oral delivery or targeting the central nervous system, for example, while trying to unload a drug cargo at the brain from an encapsulated nano-DDS in the case of *glioblastoma multiforme*—an aggressive brain tumor with a grave prognosis.^49^ Moreover, an idea of the suitability to act as a P-gp substrate also estimates whether surface grafting of a molecule will deliver a therapeutic effect in resistant cancer cells where P-gp is a major mechanism driving drug resistance.^50^

Intriguingly, the molecular descriptors can also imply the target molecules expected to bind or interact with the monomers once they are grafted on an NP. It is generally accepted that structurally similar molecules engage the same or similar targets. Hence, a molecular pair with a high Tanimoto coefficient is expected to demonstrate similar target binding. An excellent example can be the pair of SBMA and CBMA with a Tanimoto coefficient of ∼0.73. These molecules were predicted to bind similar proteins, including GPCR, phosphatase, and proteases. Similarly, both AA and DMA, with a Tanimoto coefficient of ∼0.78, were predicted to bind oxidoreductases.

The Swiss Prediction Tool assesses the macromolecular targets of a small molecule (bioactive) based on an extensive database comprising of 370,000 known bioactives on >3000 proteins from three different species, namely, humans (*H. sapiens*), house mouse (*Mus musculus*), and the brown rat (*Rattus norvegicus*). The model was validated by investigating its predictive capacity on an external test set of 500 compounds chosen randomly from the ChEMBL24 database.^37,38^ The computational tools used in the study, including the estimation of molecular similarity and target prediction, were designed on molecular structure and reactivity that remains a fundamental pillar of understanding nature. A strong validation based on experimental data has guided the development of these *in silico* platforms. Thus, the predictions were made based on the knowledge that was supported with experimental data.

The current study shows that the range of such molecular targets is diverse and includes proteins, peptides, receptors, and enzymes. It leads to an interesting discussion on choosing the right graft as the cumulative data highlight that the scope for such a decision goes beyond just drug delivery, and allied physiological aspects that a surface-engineered NP might trigger need to be considered. The range of target molecules identified here is crucial for homeostasis, and binding them with a functionalized reactive species like an NP might cause more harm than benefit, for example, by influencing unwanted drug interactions or enzymatically catalyzed reactions.

## 5 Conclusions

Gaining control over the surface chemistry of engineered NPs holds the key toward developing effective nano-DDSs. Imparting polarity by grafting hydrophilic polymeric chains, such as PEG, has emerged as an attractive strategy toward designing nano-DDSs that, apart from being effective, can also negotiate physiological barriers like mucus. However, the range of molecular candidates for such surface grafting is diverse, and performing a thorough investigation on each of them is realistically not feasible. To this end, a study comprising 14 monomers/repeating blocks of polymer chains (oxazoline, acrylamide, vinylpyrrolidone, glycerol, acryloyl morpholine, dimethyl acrylamide, hydroxypropyl methacrylamide, hydroxyethyl methacrylamide, sialic acid, carboxybetaine acrylamide, carboxybetaine methacrylate, sulfobetaine methacrylate, methacryloyloxyethyl phosphorylcholine, and vinyl-pyridinio propanesulfonate) was undertaken with the extraction of a range of molecular descriptors followed by hierarchical clustering and determination of the Tanimoto coefficient of molecular pairs. Hierarchical clustering placed the molecular pairs of oxazoline/dimethyl acrylamide, hydroxypropyl methacrylamide/hydroxyethyl methacrylamide, acrylamide/glycerol, carboxybetaine acrylamide/vinyl-pyridinio propanesulfonate, and sulfobetaine methacrylate/methacryloyloxyethyl phosphorylcholine in same clades. Similarly, the pairs of hydroxypropyl methacrylamide/hydroxyethyl methacrylamide demonstrated a Tanimoto coefficient of >0.9. A high Tanimoto coefficient of >0.8 was noted for oxazoline/vinylpyrrolidone, acrylamide/dimethyl acrylamide, acryloyl morpholine/dimethyl acrylamide, acryloyl morpholine/hydroxypropyl methacrylamide, acryloyl morpholine/hydroxyethyl methacrylamide, carboxybetaine methacrylate/sulfobetaine methacrylate, and glycerol/hydroxypropyl methacrylamide. Current *in silico* tools offer a solution to the issue by analyzing molecules and providing a range of molecular descriptors while comparing low molecular weight drug-like molecules based on structural similarity (e.g., Tanimoto coefficient). These parameters present a numerical readout that can further be used to cluster molecules based on structural similarity and physicochemical properties. Moreover, the descriptors can offer an adequate understanding of how these molecules will influence the behavior of an NP in a nano–bio interface, including information on solubility, ability to permeate the BBB, gut absorption, and molecular targets. The study, comprising a set of 14 monomers, highlighted the utility of such molecular descriptors in screening surface ligands with implications for translation.

## Supporting Information Available

Molecular descriptors obtained for the 14 monomers (XLSX)
Tanimoto coefficient matrix(XLSX)
